# A guide to selecting high-performing antibodies for TAF15 (UniProt ID: Q92804) for use in western blot, immunoprecipitation, and immunofluorescence

**DOI:** 10.12688/f1000research.160371.1

**Published:** 2025-01-06

**Authors:** Vera Ruíz Moleón, Charles Alende, Maryam Fotouhi, Riham Ayoubi, Carl Laflamme, NeuroSGC/YCharOS/EDDU collaborative group, ABIF consortium

**Affiliations:** 1 Department of Neurology and Neurosurgery, Structural Genomics Consortium, The Montreal Neurological Institute, McGill University, Montreal, Québec, H3A 2B4, Canada

**Keywords:** Q92804, TAF15, TATA-binding protein-associated factor 2N, TATA-box binding protein associated factor 15, antibody characterization, antibody validation, western blot, immunoprecipitation, immunofluorescence

## Abstract

TAF15 (TATA-box binding protein-associated factor 15) is a member of the FET protein family, known for their roles in transcriptional regulation and RNA metabolism. Here we have characterized five TAF15 commercial antibodies for western blot, immunoprecipitation, and immunofluorescence using a standardized experimental protocol based on comparing read-outs in knockout cell lines and isogenic parental controls. These studies are part of a larger, collaborative initiative seeking to address antibody reproducibility issues by characterizing commercially available antibodies for human proteins and publishing the results openly as a resource for the scientific community. While use of antibodies and protocols vary between laboratories, we encourage readers to use this report as a guide to select the most appropriate antibodies for their specific needs.

## Introduction

The FET family of RNA binding proteins includes TATA-binding protein-associated factor 2N (TAF15), Fused in Sarcoma (FUS) ^1^ and RNA binding protein EWS (EWS). TAF15 functions primarily as a transcriptional co-activator by interacting with components of the transcription machinery, including RNA polymerase II and other transcription factors, to regulate gene expression. ^2^ TAF15 contains RNA-binding motifs, enabling it to influence RNA splicing, transport, and stability. ^3^ Dysregulation or mutations in TAF15 have been implicated in certain cancers and neurodegenerative diseases, highlighting its critical role in cellular homeostasis. ^4^

This research is part of a broader collaborative initiative in which academics, funders and commercial antibody manufacturers are working together to address antibody reproducibility issues by characterizing commercial antibodies for human proteins using standardized protocols, and openly sharing the data. ^5– 7^ Here we evaluated the performance of five commercial antibodies for TAF15 for use in western blot, immunoprecipitation, and immunofluorescence, enabling biochemical and cellular assessment of TAF15 properties and function. The platform for antibody characterization used to carry out this study was endorsed by a committee of industry and academic representatives. ^8^ It consists of identifying human cell lines with adequate target protein expression and the development/contribution of equivalent knockout (KO) cell lines, followed by antibody characterization procedures using most commercially available antibodies against the corresponding protein. The standardized consensus antibody characterization protocols are openly available on Protocol Exchange, a preprint server (DOI: 10.21203/rs.3.pex-2607/v1). ^9^

The authors do not engage in result analysis or offer explicit antibody recommendations. Our primary aim is to deliver top-tier data to the scientific community, grounded in Open Science principles. This empowers experts to interpret the characterization data independently, enabling them to make informed choices regarding the most suitable antibodies for their specific experimental needs. Guidelines on how to interpret antibody characterization data found in this study are featured on the YCharOS gateway. ^10^

## Results and discussion

Our standard protocol involves comparing readouts from wild type (WT) and KO cells. ^11, 12^ The first step was to identify a cell line(s) that expresses sufficient levels of a given protein to generate a measurable signal using antibodies. To this end, we examined the DepMap (Cancer Dependency Map Portal, RRID:SCR_017655) transcriptomics database to identify all cell lines that express the target at levels greater than 2.5 log _2_ (transcripts per million “TPM” + 1), which we have found to be a suitable cut-off. ^5^ The HAP1 expresses the TAF15 transcript at 7.9, which is above the average range of cancer cells analyzed. *TAF15* KO in HAP1 cells were obtained from Horizon Discovery ( Table 1).

**Table 1.** Summary of the cell lines used.

Institution	Catalog number	RRID (Cellosaurus)	Cell line	Genotype
Horizon Discovery	C631	CVCL_Y019	HAP1	WT
Horizon Discovery	HZGHC004653c001	CVCL_TR46	HAP1	*TAF15* KO

**Figure 1.**
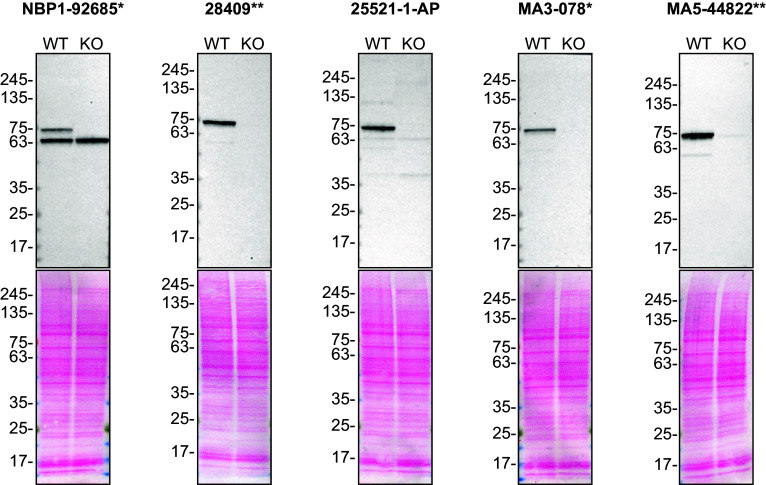
TAF15 antibody screening by western blot. Lysates of HAP1 WT and *TAF15* KO were prepared, and 30 μg of protein were processed for western blot with the indicated TAF15 antibodies. The Ponceau stained transfers of each blot are presented to show equal loading of WT and KO lysates and protein transfer efficiency from the acrylamide gels to the nitrocellulose membrane. Antibody dilutions were chosen according to the recommendations of the antibody supplier. Antibody dilution used: NBP1-92685* at 1/1000; 28409** at 1/1000; 25521-1-AP at 1/1000; MA3-078* at 1/1000; MA5-44822** at 1/1000. Predicted band size: 62 kDa. *Monoclonal antibody, **Recombinant antibody.

To screen all antibodies by western blot, WT and *TAF15* KO protein lysates were ran on SDS-PAGE, transferred onto nitrocellulose membranes, and then probed with the five TAF15 antibodies in parallel ( Figure 1).

**Figure 2.**
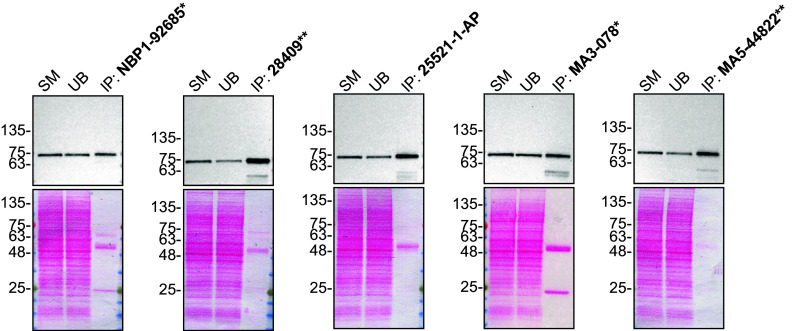
TAF15 antibody screening by immunoprecipitation. HAP1 lysates were prepared, and immunoprecipitation was performed using 1 mg of lysate and 2.0 μg of the indicated TAF15 antibodies pre-coupled to Dynabeads protein A or protein G. Samples were washed and processed for western blot with the indicated TAF15 antibody. For western blot, 28409** was used at 1/1000. The Ponceau stained transfers of each blot are shown. SM=4% starting material; UB=4% unbound fraction; IP=immunoprecipitate. *Monoclonal antibody, **Recombinant antibody.

We then assessed the capability of all five antibodies to capture TAF15 from HAP1 protein extracts using immunoprecipitation techniques, followed by western blot analysis. For the immunoblot step, a specific TAF15 antibody identified previously (refer to Figure 1) was selected. Equal amounts of the starting material (SM) and the unbound fractions (UB), as well as the whole immunoprecipitate (IP) eluates were separated by SDS-PAGE ( Figure 2).

**Figure 3.**
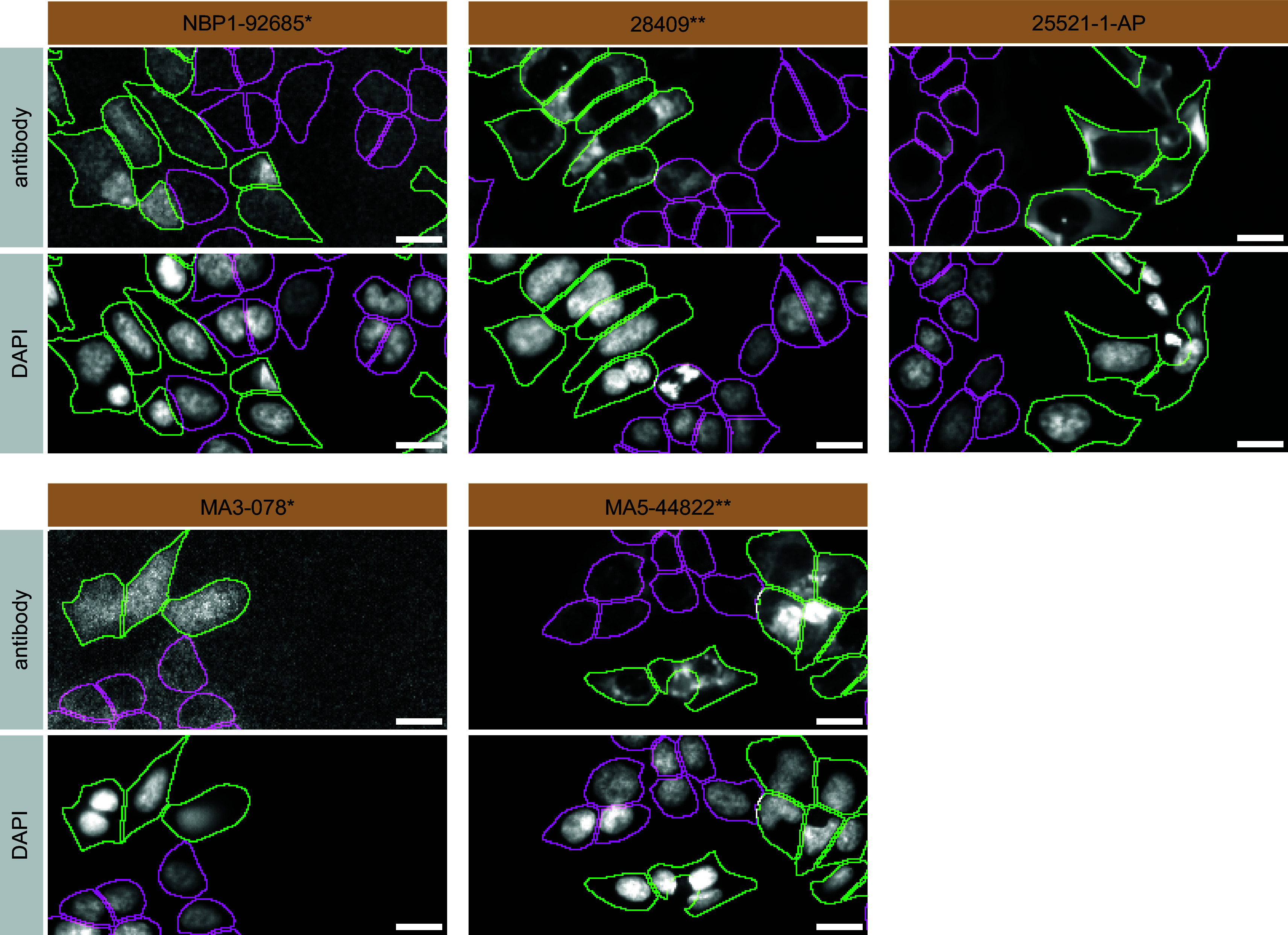
TAF15 antibody screening by immunofluorescence. HAP1 WT and *TAF15* KO cells were labelled with a green or a far-red fluorescent dye, respectively. WT and KO cells were mixed and plated to a 1:1 ratio on coverslips. Cells were stained with the indicatedTAF15 antibodies and with the corresponding Alexa-fluor 555 coupled secondary antibody including DAPI. Acquisition of the blue (nucleus-DAPI), green (WT), red (antibody staining) and far-red (KO) channels was performed. Representative images of the merged blue and red (grayscale) channels are shown. WT and KO cells are outlined with green and magenta dashed line, respectively. When an antibody was recommended for immunofluorescence by the supplier, we tested it at the recommended dilution. The rest of the antibodies were tested at 1 and 2 μg/ml and the final concentration was selected based on the detection range of the microscope used and a quantitative analysis not shown here. Antibody dilution used: NBP1-92685* at 1/1000; 28409** at 1/150; 25521-1-AP at 1/50; MA3-078* at 1/500; MA5-44822** at 1/1000. Bars = 10 μm. *Monoclonal antibody, **Recombinant antibody.

For immunofluorescence, the five antibodies were screened using a mosaic strategy. First, HAP1 WT and *TAF15* KO cells were labelled with different fluorescent dyes in order to distinguish the two cell lines, and the TAF15 antibodies were evaluated. Both WT and KO lines imaged in the same field of view to reduce staining, imaging and image analysis bias ( Figure 3). Quantification of immunofluorescence intensity in hundreds of WT and KO cells was performed for each antibody tested, and the images presented in Figure 3 are representative of this analysis. ^9^

In conclusion, we have screened five TAF15 commercial antibodies by western blot, immunoprecipitation, and immunofluorescence by comparing the signal produced by the antibodies in human HAP1 WT and *TAF15* KO cells. To assist users in interpreting antibody performanyce, Table 3 outlines various scenarios in which antibodies may perform in all three applications. ^5^ Several high-quality and renewable antibodies that successfully detect TAF15 were identified in all applications. Researchers who wish to study TAF15 in a different species are encouraged to select high-quality antibodies, based on the results of this study, and investigate the predicted species reactivity of the manufacturer before extending their research.

The underlying data listed below for this study can be found on Zenodo, an open-access repository for which YCharOS has its own collection of antibody characterization reports.

### Limitations

Inherent limitations are associated with the antibody characterization platform used in this study. Firstly, the YCharOS project focuses on renewable (recombinant and monoclonal) antibodies and does not test all commercially available TAF15 antibodies. YCharOS partners provide approximately 80% of all renewable antibodies, but some top-cited polyclonal antibodies may not be available through these partners.

Secondly, the YCharOS effort employs a non-biased approach that is agnostic to the protein for which antibodies have been characterized. The aim is to provide objective data on antibody performance without preconceived notions about how antibodies should perform or the molecular weight that should be observed in western blot. As the authors are not experts in TAF15 only a brief overview of the protein's function and its relevance in disease is provided. TAF15 experts are invited to analyze and interpret observed banding patterns in western blots and subcellular localization in immunofluorescence.

Thirdly, YCharOS experiments are not performed in replicates primarily due to the use of multiple antibodies targeting various epitopes. Once a specific antibody is identified, it validates the protein expression of the intended target in the selected cell line, confirms the lack of protein expression in the KO cell line and supports conclusions regarding the specificity of the other antibodies. All experiments are performed using master mixes, and meticulous attention is paid to sample preparation and experimental execution. In IF, the use of two different concentrations serves to evaluate antibody specificity and can aid in assessing assay reliability. In instances where antibodies yield no signal, a repeat experiment is conducted following titration. Additionally, our independent data is performed subsequently to the antibody manufacturers internal validation process, therefore making our characterization process a repeat.

Lastly, as comprehensive and standardized procedures are respected, any conclusions remain confined to the experimental conditions and cell line used for this study. The use of a single cell type for evaluating antibody performance poses as a limitation, as factors such as target protein abundance significantly impact results. ^9^ Additionally, the use of cancer cell lines containing gene mutations poses a potential challenge, as these mutations may be within the epitope coding sequence or other regions of the gene responsible for the intended target. Such alterations can impact the binding affinity of antibodies. This represents an inherent limitation of any approach that employs cancer cell lines.

## Method

The standardized protocols used to carry out this KO cell line-based antibody characterization platform was established and approved by a collaborative group of academics, industry researchers and antibody manufacturers. ^8^ The detailed materials and step-by-step protocols used to characterize antibodies in western blot, immunoprecipitation and immunofluorescence are openly available on Protocol Exchange, a preprint server (DOI: 10.21203/rs.3.pex-2607/v1). ^9^ Brief descriptions of the experimental setup used to carry out this study can be found below.

### Cell lines and antibodies

Cell lines used and primary antibodies tested in this study are listed in Tables 1 and 2, respectively. To ensure that the cell lines and antibodies are cited properly and can be easily identified, we have included their corresponding Research Resource Identifiers, or RRID. ^13, 14^

**Table 2.** Summary of the TAF15 antibodies tested.

Company	Catalog number	Lot number	RRID (Antibody Registry)	Clonality	Clone ID	Host	Concentration (μg/μL)	Vendors recommended applications
Bio-Techne	NBP1-92685 *	102313	AB_11037253	monoclonal	4D71	mouse	1	Wb, IF
Cell Signaling Technology	28409 **	1	AB_2798957	recombinant mono	D8V6Q	rabbit	0.146	Wb, IP
Proteintech	25521-1-AP	41674	AB_2880116	polyclonal	-	rabbit	2.66	Wb
Thermo Fisher Scientific	MA3-078 *	WL343399	AB_2633323	monoclonal	8TA-2B10	mouse	n/a	Wb, IP, IF
Thermo Fisher Scientific	MA5-44822 **	YE3913388B	AB_2931279	recombinant mono	JE61-92	rabbit	1	Wb, IF

Wb=western blot; IF=immunofluorescence; IP=immunoprecipitation.

* Monoclonal antibody.

** Recombinant antibody, n/a=not available.

**Table 3.** Illustrations to assess antibody performance in all western blot, immunoprecipitation and immunofluorescence.

Western blot	Immunoprecipitation	Immunofluorescence
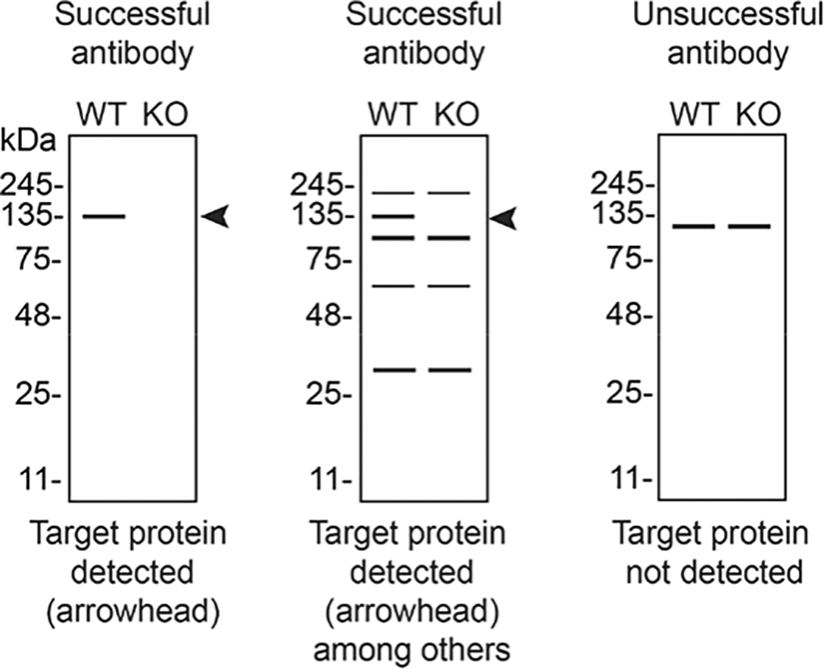	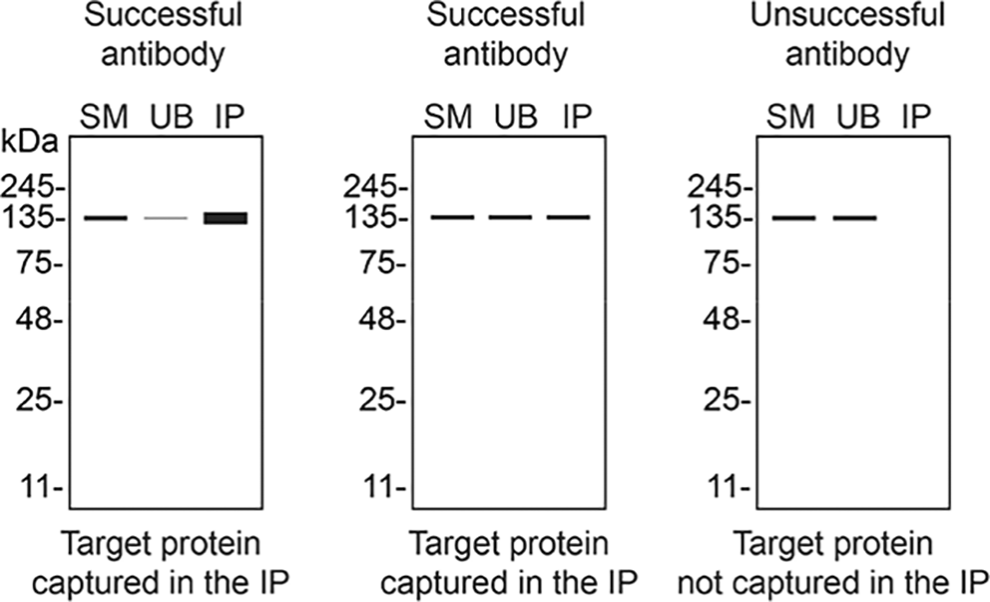	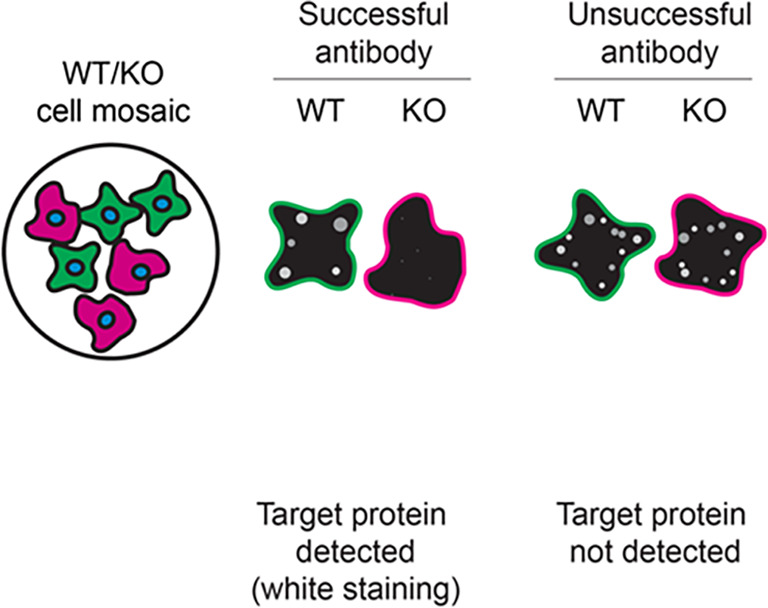

This table has been reproduced with permission from Ayoubi et al., Elife, 2023. ^5^

Peroxidase-conjugated goat anti-rabbit and anti-mouse antibodies are (Thermo Fisher Scientific, cat. number 65-6120 and 62-6520). Alexa-555-conjugated goat anti-rabbit and anti-mouse secondary antibodies (Thermo Fisher Scientific, cat. number A-21429 and A-21424). Peroxidase-conjugated Protein A for IP detection is from MilliporeSigma, cat. number P8651.

### Antibody screening by western blot

HAP1 WT and *TAF15* KO cells were collected in RIPA buffer (25 mM Tris-HCl pH 7.6, 150 mM NaCl, 1% NP-40, 1% sodium deoxycholate, 0.1% SDS) (Thermo Fisher Scientific, cat. number 89901) supplemented with 1× protease inhibitor cocktail mix (MilliporeSigma, cat. number P8340). Lysates were sonicated briefly and incubated 30 min on ice. Lysates were spun at ~110,000 *× g* for 15 min at 4°C and equal protein aliquots of the supernatants were analyzed by SDS-PAGE and western blot. BLUelf prestained protein ladder (GeneDireX, cat. number PM008-0500) was used.

Western blots were performed with precast midi 4-20% Tris-Glycine polyacrylamide gels (Thermo Fisher Scientific, cat. number WXP42012BOX) ran with Tris/Glycine/SDS buffer (Bio-Rad, cat. number 1610772), loaded in Laemmli loading sample buffer (Thermo Fisher Scientific, cat. number AAJ61337AD) and transferred on nitrocellulose membranes. Proteins on the blots were visualized with Ponceau S staining (Thermo Fisher Scientific, cat. number BP103-10) which is scanned to show together with individual western blot. Blots were blocked with 5% milk for 1 hr, and antibodies were incubated O/N at 4°C with 5% milk in TBS with 0.1% Tween 20 (TBST) (Cell Signalling Technology, cat. number 9997). Following three washes with TBST, the peroxidase conjugated secondary antibody was incubated at a dilution of ~0.2 μg/ml in TBST with 5% milk for 1 hr at room temperature followed by three washes with TBST. Membranes were incubated with Pierce ECL (Thermo Fisher Scientific, cat. number 32106) prior to detection with the iBright™ CL1500 Imaging System (Thermo Fisher Scientific, cat. number A44240).

### Antibody screening by immunoprecipitation

Antibody-bead conjugates were prepared by adding 2 μg of antibody to 500 μl of Pierce IP Lysis Buffer from Thermo Fisher Scientific (cat. number 87788) in a microcentrifuge tube, together with 30 μl of Dynabeads protein A- (for rabbit antibodies) or protein G- (for mouse antibodies) (Thermo Fisher Scientific, cat. number 10002D and 10004D, respectively). 10 μl of antibodies 28409** and MA3-078* were used in the IP. Tubes were rocked for ~1 hr at 4°C followed by two washes to remove unbound antibodies.

HAP1 WT were collected in Pierce IP buffer (25 mM Tris-HCl pH 7.4, 150 mM NaCl, 1 mM EDTA, 1% NP-40 and 5% glycerol) supplemented with protease inhibitor. Lysates were rocked 30 min at 4°C and spun at 110,000 *× g* for 15 min at 4°C. 0.5 ml aliquots at 2 mg/ml of lysate were incubated with an antibody-bead conjugate for ~1 h at 4°C. The unbound fractions were collected, and beads were subsequently washed three times with 1.0 ml of IP buffer and processed for SDS-PAGE and western blot on precast midi 4-20% Tris-Glycine polyacrylamide gels. Protein A:HRP was used as a secondary detection system at a concentration of 0.3 μg/ml.

### Antibody screening by immunofluorescence

HAP1 WT and *TAF15* KO cells were labelled with a green and a far-red fluorescence dye, respectively (Thermo Fisher Scientific, cat. number C2925 and C34565). The nuclei were labelled with DAPI (Thermo Fisher Scientific, cat. Number D3571) fluorescent stain. WT and KO cells were plated on 96-well plate with optically clear flat-bottom (Perkin Elmer, cat. number 6055300) as a mosaic and incubated for 24 hrs in a cell culture incubator at 37 ^o^C, 5% CO _2_. Cells were fixed in 4% paraformaldehyde (PFA) (VWR, cat. number 100503-917) in phosphate buffered saline (PBS) (Wisent, cat. number 311-010-CL). Cells were permeabilized in PBS with 0,1% Triton X-100 (Thermo Fisher Scientific, cat. number BP151-500) for 10 min at room temperature and blocked with PBS with 5% BSA, 5% goat serum (Gibco, cat. number 16210-064) and 0.01% Triton X-100 for 30 min at room temperature. Cells were incubated with IF buffer (PBS, 5% BSA, 0.01% Triton X-100) containing the primary TAF15 antibodies overnight at 4°C. Cells were then washed 3 × 10 min with IF buffer and incubated with corresponding Alexa Fluor 555-conjugated secondary antibodies in IF buffer at a dilution of 1.0 μg/ml for 1 hr at room temperature with DAPI. Cells were washed 3 × 10 min with IF buffer and once with PBS.

Images were acquired on an ImageXpress micro confocal high-content microscopy system (Molecular Devices), using a 20x NA 0.95 water immersion objective and scientific CMOS cameras, equipped with 395, 475, 555 and 635 nm solid state LED lights (lumencor Aura III light engine) and bandpass filters to excite DAPI, Cellmask Green, Alexa-555 and Cellmask Red, respectively. Images had pixel sizes of 0.68 x 0.68 microns, and a z-interval of 4 microns. For analysis and visualization, shading correction (shade only) was carried out for all images. Then, maximum intensity projections were generated using 3 z-slices. Segmentation was carried out separately on maximum intensity projections of Cellmask channels using CellPose 1.0, and masks were used to generate outlines and for intensity quantification. ^15^ Figures were assembled with Adobe Illustrator.

## Data Availability

Zenodo: Antibody Characterization Report for TAF15, https://doi.org/10.5281/zenodo.10085319. ^16^ Zenodo: Dataset for the TAF15 antibody screening study, https://doi.org/10.5281/zenodo.14536805 ^17^ Data are available under the terms of the Creative Commons Attribution 4.0 International license (CC-BY 4.0).

## References

[1] Alshalfie W Fotouhi M Ayoubi R : The identification of high-performing antibodies for RNA-binding protein FUS for use in Western Blot, immunoprecipitation, and immunofluorescence. *F1000Res.* 2023;12:376. 10.12688/f1000research.133220.2 37384305 PMC10293799

[2] Bertolotti A Lutz Y Heard DJ : hTAF (II)68, a novel RNA/ssDNA-binding protein with homology to the pro-oncoproteins TLS/FUS and EWS is associated with both TFIID and RNA polymerase II. *EMBO J.* 1996;15(18):5022–5031. 10.1002/j.1460-2075.1996.tb00882.x 8890175 PMC452240

[3] Schwartz JC Cech TR Parker RR : Biochemical Properties and Biological Functions of FET Proteins. *Annu. Rev. Biochem.* 2015;84:355–379. 10.1146/annurev-biochem-060614-034325 25494299 PMC9188303

[4] Johnson CN Sojitra KA Sohn EJ : Insights into Molecular Diversity within the FUS/EWS/TAF15 Protein Family: Unraveling Phase Separation of the N-Terminal Low-Complexity Domain from RNA-Binding Protein EWS. *J. Am. Chem. Soc.* 2024;146(12):8071–8085. 10.1021/jacs.3c12034 38492239 PMC11156192

[5] Ayoubi R Ryan J Biddle MS : Scaling of an antibody validation procedure enables quantification of antibody performance in major research applications. *elife.* 2023;12. 10.7554/eLife.91645 PMC10666931 37995198

[6] Carter AJ Kraemer O Zwick M : Target 2035: probing the human proteome. *Drug Discov. Today.* 2019;24(11):2111–2115. 10.1016/j.drudis.2019.06.020 31278990

[7] Licciardello MP Workman P : The era of high-quality chemical probes. *RSC Med. Chem.* 2022;13(12):1446–1459. 10.1039/D2MD00291D 36545432 PMC9749956

[8] Ayoubi R Ryan J Gonzalez Bolivar S : A consensus platform for antibody characterization. *Nat. Protoc.* 2024. 10.1038/s41596-024-01095-8 39690206 PMC13054593

[9] Ayoubi R Ryan J Bolivar SG : A consensus platform for antibody characterization (Version 1). *Protocol Exchange.* 2024. 10.1038/s41596-024-01095-8 PMC13054593 39690206

[10] Biddle MS Virk HS : YCharOS open antibody characterisation data: Lessons learned and progress made. *F1000Res.* 2023;12(12):1344. 10.12688/f1000research.141719.1 37854875 PMC10579855

[11] Laflamme C McKeever PM Kumar R : Implementation of an antibody characterization procedure and application to the major ALS/FTD disease gene C9ORF72. *elife.* 2019;8:8. 10.7554/eLife.48363 PMC6794092 31612854

[12] Alshafie W Fotouhi M Shlaifer I : Identification of highly specific antibodies for Serine/threonine-protein kinase TBK1 for use in immunoblot, immunoprecipitation and immunofluorescence. *F1000Res.* 2022;11:977. 10.12688/f1000research.124632.1 36415206 PMC9647147

[13] Bandrowski A Pairish M Eckmann P : The Antibody Registry: ten years of registering antibodies. *Nucleic Acids Res.* 2023;51(D1):D358–D367. 10.1093/nar/gkac927 36370112 PMC9825422

[14] Bairoch A : The Cellosaurus, a Cell-Line Knowledge Resource. *J. Biomol. Tech.* 2018;29(2):25–38. 10.7171/jbt.18-2902-002 29805321 PMC5945021

[15] Stringer C Wang T Michaelos M : Cellpose: a generalist algorithm for cellular segmentation. *Nat. Methods.* 2021;18(1):100–106. 10.1038/s41592-020-01018-x 33318659

[16] Ruíz Moléon V Fotouhi M Alende C : Antibody Characterization Report for TATA-binding protein associated factor 2N (TAF15). 2023. 10.5281/zenodo.10085319

[17] Laflamme C : Dataset for the TAF15 antibody screening study.[Dataset]. *Zenodo.* 2024. 10.5281/zenodo.14536805

