# Supplementary material for: A guide to selecting high-performing antibodies for TAF15 (UniProt ID: Q92804) for use in western blot, immunoprecipitation, and immunofluorescence
Source: F1000Res. 2025 Jan 6;14:37. [Version 1] doi: 10.12688/f1000research.160371.1 (PMC11966097; doi:10.12688/f1000research.160371.1)
Supplement: Peer review history

# Reviewer report: Reviewer response for version 1

**DOI:** 10.5256/f1000research.176259.r370782

**Published:** 2025-04-02

**Authors:** Sian E. Evans, Carolyn Jones

**Affiliations:** 1 University of Leicester, Leicester, England, UK

This article offers a clear and straightforward demonstration of the specificity of several antibodies targeting the protein TAF15. It utilises three techniques—Western Blotting (WB), Immunoprecipitation (IP), and Immunofluorescence (IF)—with all protocols readily accessible for researchers to replicate. Table 3 presents example results, which are incredibly helpful for interpreting the data. Finally, adding a limitations section provides valuable insights to inspire further reflection during the planning and execution of such experiments.

Are sufficient details of methods and materials provided to allow replication by others?

Yes

Is the rationale for creating the dataset(s) clearly described?

Yes

Are the datasets clearly presented in a useable and accessible format?

Yes

Are the protocols appropriate and is the work technically sound?

Yes

Reviewer Expertise:

Cell culture (primary cells and cell lines), patient-derived explants (PDE), Western blotting,  Immunohistochemistry (IHC),  immunofluorescence (IF),  qPCR, RNA/DNA/protein purification

We confirm that we have read this submission and believe that we have an appropriate level of expertise to confirm that it is of an acceptable scientific standard.

# Reviewer report: Reviewer response for version 1

**DOI:** 10.5256/f1000research.176259.r370781

**Published:** 2025-04-01

**Authors:** Deborah Moshinsky

**Affiliations:** 1 Institute for Protein Innovation, Boston, Massachusetts, USA

The authors describe Western Blot, Immunoprecipitation, and Immunofluorescence testing results for 5 commercially available antibodies against TATA-box binding protein-associated factor 15 (TAF15).  Clear methods were given and referenced, and the results were clearly delineated in figures and tables.  A cartoon figure was also shown to assist in result interpretation.

 Brief information was given about the biology of the TAF15.  The authors adequately describe that they are not experts in the biology of the target, so this is understandable.  However, in light of the immunofluorescence data where some antibodies are detecting a cytoplasmic protein and others a protein in the nucleus, a mention about the expected cellular localization of the target would have been welcome, or information about the current thoughts in the field about localization.

Are sufficient details of methods and materials provided to allow replication by others?

Yes

Is the rationale for creating the dataset(s) clearly described?

Yes

Are the datasets clearly presented in a useable and accessible format?

Yes

Are the protocols appropriate and is the work technically sound?

Yes

Reviewer Expertise:

Antibody characterization and validation

I confirm that I have read this submission and believe that I have an appropriate level of expertise to confirm that it is of an acceptable scientific standard.
